# Rational Design of Plant Hairpin-like Peptide EcAMP1: Structural–Functional Correlations to Reveal Antibacterial and Antifungal Activity

**DOI:** 10.3390/molecules27113554

**Published:** 2022-05-31

**Authors:** Anna S. Barashkova, Dmitry Y. Ryazantsev, Eugene A. Rogozhin

**Affiliations:** 1 Shemyakin-Ovchinnikov Institute of Bioorganic Chemistry, Russian Academy of Natural Sciences (RAS), ul. Miklukho-Maklaya, 16/10, 117997 Moscow, Russia; barashkova.an@gmail.com (A.S.B.); d.yu.ryazantsev@gmail.com (D.Y.R.); 2 Gause Institute of New Antibiotics, ul. Bolshaya Pirogovskaya, 11, 119021 Moscow, Russia

**Keywords:** hairpin-like peptides, plant antimicrobial peptides, antifungal activity, amino acid substitution

## Abstract

Plant antimicrobial peptides from the α-hairpinins family (hairpin-like peptides) are known to possess a wide range of biological activities. However, less is known about the structural determinants of their antimicrobial activity. Here, we suggest that spatial structure as well as surface charge and hydrophobicity level contribute to the antimicrobial properties of α-hairpinin EcAMP1 from barnyard grass (*Echinochloa cruss-galli*) seeds. To examine the role of the peptide spatial structure, two truncated forms of EcAMP1 restricted by inner and outer cysteine pairs were synthesized. It was shown that both truncated forms of EcAMP1 lost their antibacterial activity. In addition, their antifungal activity became weaker. To review the contribution of surface charge and hydrophobicity, another two peptides were designed. One of them carried single amino acid substitution from tryptophan to alanine residue at the 20th position. The second one represented a truncated form of the native EcAMP1 lacking six C-terminal residues. But the α-helix was kept intact. It was shown that the antifungal activity of both modified peptides weakened. Thereby we can conclude that the secondary structural integrity, hydrophobic properties, and surface charge all play roles in the antimicrobial properties of α-hairpinins. In addition, the antibacterial activity of cereal α-hairpinins against Gram-positive bacteria was described for the first time. This study expands on the knowledge of structure–function interactions in antimicrobial α-hairpinins.

## 1. Introduction

Plants produce a huge amount of different biologically active compounds. Some of them participate in ontogenesis; several molecules are known to mediate plant signaling and interaction [1]. A large group of compounds is engaged in plant defense and plant–microbe interaction [2,3]. These molecules build plant innate immunity. The group of plant defense peptides is of particular interest. Plant defense peptides represent a very special group of metabolites that are synthesized constitutively. They provide a so-called “first defense line” against a variety of environmental stress factors, primarily of a biotic nature [4,5,6]. Plant defense peptides are divided into seven families according to their Cys motif [7,8]. Hairpin-like peptides (α-hairpinins) are one of the youngest structural families. α-hairpinins have four Cys residues that form two disulfide bonds. Their structure is represented by a helix-turn-helix motif. Despite the high similarity of spatial structure, α-hairpinins possess low sequence homology and surprisingly high functional diversity [9]. Peptides from *Fagopurum esculentum*, *Veronica hederifolia,* and *Cucurbita maxima* inhibit serine proteinases by entering the enzyme active center [10,11,12]. Peptides from *Luffa cyllindrica* disrupt protein biosynthesis via ribosome inactivation [13]. Peptides from *Zea mays*, *Stellaria media*, *Triticum kiharae,* and *Echinochloa crus-galli* suppress the growth of bacteria and filamentous fungi [14,15,16,17]. The mechanism of antimicrobial activity is still unclear. However, a few facts are already known. First of all, EcAMP1 an antifungal α-hairpinin from barnyard grass (*E. cruss-galli*) seeds, is known to internalize inside fungal conidia without plasma membrane disruption [18]. Amino acid substitution Pro19Hyp leads to a decrease in antifungal activity and carbohydrate binding [19]. In addition, the loss of C-terminal random coil in EcAMP2 leads to a complete loss of peptide activity [15].

In addition, another *E. crus-galli* homologous α-hairpinin EcAMP2 represents a “naturally designed” variant of EcAMP1 lacking the C-terminal random coil. This modification led to a complete loss of antifungal activity of EcAMP2 [15]. It became a piece of evidence of spatial structure contribution significance in the activity of the peptide.

The modification of the peptide structure is widely applied in drug design. The main purpose of such findings is to find molecules with enchased activity and reduced toxicity [20,21]. Another purpose is to specify peptides to a particular target or to functionalize peptide molecules [22,23,24]. Computational mining, prediction of target properties [25] and chemical and microbiological synthesis [20,26] are being used in peptide design.

Here, we applied rational design to bring out structural determinants, which are responsible for antifungal activity of α-hairpinin EcAMP1. First of all, and encouraged by an example of natural EcAMP2, we used chemically synthesized truncated analogs to estimate the N-terminal and C-terminal random coils as well as disulfide bond integrity on antifungal properties of peptides. The second part of the research covered the role of single amino acid substitution in the β-hairpin site and contribution of the total surface charge in EcAMP1 antifungal activity.

## 2. Results and Discussion

**Figure 1:**
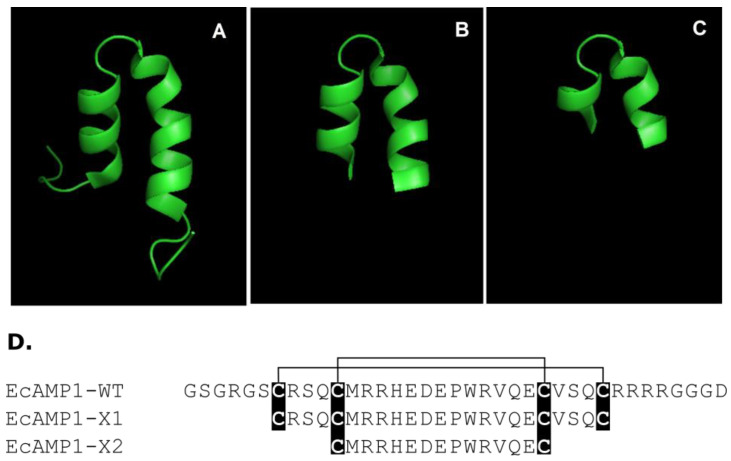
Structures of the wild-type EcAMP1 and its truncated synthetic analogs EcAMP1-X1 and EcAMP1-X2. Modeling of spatial structure of wild-type EcAMP1-WT (**A**); truncated form of EcAMP1-X1 restricted up to outer disulfides (**B**); truncated form of EcAMP1-X2 restricted up to inner disulfides (**C**); multiple alignment of EcAMP1-WT, EcAMP1-X1, and EcAMP1-X2 (**D**); Cys residues are shown in black boxes, and disulfide bridges are shown as black lines above.

This study presents the first attempt to show a rational design of plant defense peptides that belong to the α-hairpinins previously isolated from wild cereals to reveal so-called minimal structural basis, which is needed for the demonstration of biological activity (antibacterial and antifungal). α-hairpinin EcAMP1 from barnyard grass (*E. crus-galli*) seeds was selected for a model because this peptide revealed significant and specific antifungal effects according to a test panel of phytopathogenic fungi and oomycetes in vitro (so, inhibition activity was registered for 11 from 15 microbes) [27]. As mentioned above, the 3D-structure for EcAMP1 was determined before; thereby, this fact was key for the structure–function correlation based on predominantly spatial–molecular conformation (the structure was previously deposited to the Protein Data Bank, ID: 2L2R). Furthermore, another example is the hairpin-like peptide Tk-AMP-X2 isolated from wheat (*Triticum kiharae*) that possess antifungal activity relative to obligate parasites from maize (*Zea mays*) [14]. Interestingly, this peptide displayed high spatial homology with blockers of voltage-gated potassium channels from scorpion venom, and thus, is a background for site-specific mutagenesis to generate peptide artificial analogues [23,24]. To minimize the polypeptide chain, two mutant variants of the EcAMP1 were suggested: firstly, with truncated N- and C- termini restricted by Cys1–Cys4 (EcAMP1-X1) and, secondly, with potentially truncated both α-helices restricted by Cys2–Cys3 (EcAMP1-X2) (Figure 1A). Two of these peptides were obtained by solid-phase peptide synthesis, and the controlling of a correct folding was performed by analytical reversed-phase HPLC, MALDI-TOF MS, and CD spectroscopy (see Supplemental Data, Figures S1–S5).

Antimicrobial testing of all the modified EcAMP1 peptides compared with the native form was conducted according to a line of collected Gram-positive and Gram-negative bacterial strains, as well as yeasts, at a wide range of active concentrations (0.625–80 µM). A linear α-helical antimicrobial peptide cathelicidin (LL37) was applied as the positive control [28] (see Supplemental Data, Tables S1–S4). It was shown that the native EcAMP1 (EcAMP1-WT) was inactive against Gram-negative *E. coli* and *P. aeruginosa*. At the same time, EcAMP1-WT demonstrated distinguishable antibacterial activity on Gram-positive *S. aureus* at 5 µM (IC_50_). Interestingly, EcAMP1-WT demonstrated a weaker antibacterial activity in comparison with its closest homologue (~63%), MBP-1 from maize (*Zea mays*) kernels, with a significant effect against bacterial cultures (IC_50_~0.25–2.5 µM) [17].

Previously, it was shown that EcAMP1 inhibits the growth of phytopathogenic fungi and oomycetes [27]. Here, it was found that this peptide can also inhibit opportunistic yeast *C. albicans* at 0.625 µM (IC_50_) and almost completely suppressed it at 1.25 µM (MIC_99_). Thus, yeasts are probably more susceptible to EcAMP1 than filamentous fungi and oomycetes (IC_50_ ~4.5–18.2 µM) [27].

In contrast to the native form, both truncated peptides (EcAMP1-X1 and EcAMP1-X2) were absolutely inactive against all microbes tested at concentrations up to 80 µM. Consequently, the results obtained confirm the hypothesis: the shortening of the N- and C-termini led to the disruption of the secondary structure forming (α-helices) and influences correct folding.

In the case of the activity of EcAMP-X1 and EcAMP-X2 on phytopathogenic filamentous fungi, it was shown that both peptides maintained the activity against most fungi, but their IC50 levels were higher. This illustrates the decrease in antifungal activity. In addition, the fungistatic effect on *Fusarium* spp. was detected. This maybe appeared in peptide selectivity (Table 1). Based on the comparative antimicrobial testing of the native α-hairpinin EcAMP1 and its two truncated forms, we can draw a conclusion about the critical suppression of the biological action for peptides with a disturbance of secondary structural elements.

These data correlate with previous results obtained on a truncated form of the Sm-AMP-X, another α-hairpinin from common chickweed (*Stellaria media*). The antifungal activity loss was detected as a consequence of secondary structure building disturbance and complete elimination of the N-terminal and C-terminal fragments that were precisely involved in the realization of antifungal activity [29].

**Figure 2:**
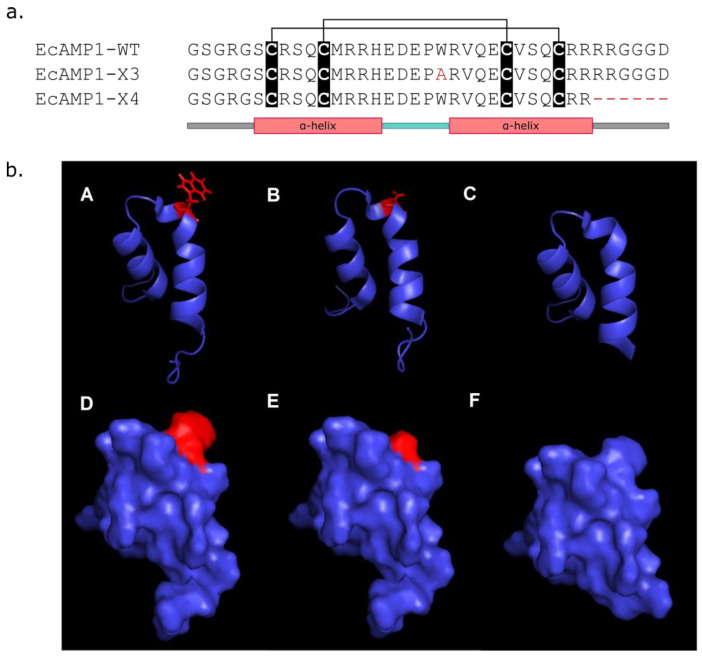
Structures of the wild-type EcAMP1 and its recombinant analogs EcAMP1-X3 and EcAMP1-X4. (**a**) Amino acid sequences of peptides compared to a schematic view of α-hairpinin secondary structure. Cys residues are shown in black boxes, and disulfide bridges are shown as black lines above. EcAMP1-X3 sequence Ala that substituted Trp 20 is marked in red. EcAMP1-X4 lacking C-terminal residues are marked by red gaps. (**b**) 3D modeling of wild-type EcAMP1 and its modified recombinant analogs. A–C ribbon models and D–F surface models. Wild-type EcAMP1-WT (**A**,**D**); EcAMP1-X3 (**B**,**E**); and EcAMP1-X4 (**C**,**F**). Trp20 in EcAMP1-WT and Ala20 in EcAMP-X3 are shown in red.

In the second part of this study, a site-specific mutagenesis of the EcAMP1 structure was carried out. According to the β-hairpin amino acid residue side chain spatial orientation, it should be hypothesized that they are involved in the binding with the cell wall and plasma membrane components [27]. In addition, EcAMP1 peptide is positively charged at a neutral pH, so an investigation as to whether a positive charge plays a key role in the activity of plant α-hairpinins seems to be important, whereas some of them are anionic [10,12]. It was also shown that EcAMP2 lacking five C-terminal residues does not possess antibacterial activity. Therefore, two modified EcAMP1 recombinant analogs (EcAMP1-X3 and EcAMP1-X4) were produced by heterologous expression in the *E. coli* system (Figure 2, see Supplemental Data, Figure S6). EcAMP1-X3 carries one amino acid substitution Trp20Ala, and EcAMP1-X4 represents a truncated form of EcAMP1-WT lacking six C-terminal residues. The yield of peptides was 4.5 mg/L for EcAMP1-WT and 2.3 and 0.9 mg/L for EcAMP1-X3 and EcAMP1-X4, respectively. Both forms were tested against phytopathogenic fungi from the genus *Fusarium* in compare with the wild-type peptide.

It was shown that both modifications led to a significant decrease in antifungal activity compared to the wild-type EcAMP1 (Table 2, see Supplemental Data, Figure S7).

An initial mechanism of EcAMP1 antifungal activity was achieved by the hydrophobic interaction of the side chain of tryptophan residue with fungal membrane. The substitution of Trp20 with Ala in the EcAMP1-X3 structure probably led to a significant disturbance of such interaction and a decrease in peptide activity. In addition, proteins and peptides are potentially able to bind with hydrophobins that are located on the surface of fungal conidia [30,31]. In that case, fungal hydrophobins might be firstly considered as molecular targets for an AMP attack.

Changes in the peptide binding properties also result in a space reorientation of the second α-helical domain (Figure 2C,F). Interestingly, the mutation of Trp/Ala in the MBP1 structure led to a complete inactivation against *E. coli* [32]. This confirms a critical role of tryptophan residue located in the β-hairpin in antifungal and antibacterial activity. Moreover, the unusual natural form of the EcAMP1 peptide consisting of a substitution from proline to hydroxyproline at the 19th position demonstrated a weaker activity against a model plant pathogenic fungus *F. solani* [19]. In the case of EcAMP1-X4, the removal of six C-terminal amino acid residues concerns the second α-helix and might lead to a destabilization of the molecule structure. Thus, the forming of all secondary structural elements in α-hairpinin is quite important for biological activity.

## 3. Materials and Methods

### 3.1. Microorganisms

#### 3.1.1. Bacteria and Yeast

Gram-positive bacterium *Staphylococcus aureus*, Gram-negative bacteria *Escherichia coli*, *Pseudomonas auroginosa*, and yeast *Candida albicans* were located in the Collection of Division of Pharmacognosy, Department of Medicinal Chemistry, Uppsala University, Sweden.

#### 3.1.2. Plant Pathogenic Filamentous Fungi

*Fusarium graminearum* VKM F-1668, *Aspergillus niger* VKM F-33, and *Bipolaris sorokiniana* VKM F-1446 were purchased in All-Russian Collection of Microorganisms G.K. Skryabin Institute Biochemistry and Physiology of Microorganisms Russian Academy of Sciences (Pushchino, Moscow region, Russia); *F. oxysporum* strain TSKHA-4. *F. solani* and *Alternaria alternata* were isolated from damaged potato plants and were kindly supplied by the Department of Plant Protection K.A Timiryazev Russian State Agrarian University (Moscow, Russia).

### 3.2. Design of the EcAMP1 Analogs

For these experiments, four modified EcAMP1 analogs were designed to reveal structure–function relationships in antimicrobial activity (Table 3).

### 3.3. Solid-Phase Peptide Synthesis

Chemical synthesis of the truncated EcAMP1-X1 and EcAMP1-X2 was carried out as described earlier [19] with modifications [33]. Briefly, solid-phase peptide synthesis was performed on an automatic peptide synthesizer (Agilent Technologies, Santa Clara, CA, USA) based on the Gilson automated liquid handler system (Gilson Scientific Ltd., Dunstable, UK), according to Gilson application note 228 (Gilson Scientific Ltd., Dunstable, UK). The peptides were eluted with a linear H_2_O–MeCN gradient (from 5% to 35% of MeCN), with 0.1% trifluoroacetic acid, at a flow rate of 30 mL/min. A polystyrene-PEG 2000 block-copolymer resin (Sigma-Aldrich, St. Louis, MO, USA), modified with carboxy-trytil linker (Tentagel HL-TRT, Rapp Polymere, Tübingen, Germany) and Fmoc-protected amino acids from Iris Biotech (Marktredwitz, Germany) were used, except for Fmoc-Hyp(tBu)-OH, which was from Novabiochem (Sigma-Aldrich, St. Louis, MO, USA). Acetyl chloride, 4-methyl piperidine, diisopropylethylamine, sym-collidine, and HATU were from Acros Organics (Morris, NJ, USA) and Sigma-Aldrich (St. Louis, MO, USA), respectively. C-terminal amino acid was attached to the Ac-Cl-activated resin, in the presence of Hunig’s base for 2 h. Peptide assembly was performed by Fmoc methodology, using HATU/collidine activation. An 8-fold excess of amino acids was used within 30 min of the condensation time. After the synthesis, the protected peptidyl polymer was washed with diethyl ether, then dried, and treated with trifluoroacetic acid/dithiothreitol/deionized water/triispropylsilane (TFA/DTT/H_2_O/TIS) 150/4/3/0.5 (weight proportion) mixture. Fifteen milliliters of the mixture was applied to 1 g of peptidyl polymer for 2 h. Then the solution was filtered out, and the dry peptide was precipitated with a ten-fold volume of diethyl ether and kept at 4 °C for 8 h. The precipitated peptides were centrifuged, washed three times with diethyl ether, and then dried under vacuum. Crude peptide was purified by HPLC and then lyophilized. Pure linear peptides were dissolved in 50 mM of ammonium bicarbonate in water/acetonitrile 90:10 to a final concentration of 0.5 mg/mL. The resulting solution was stirred in air overnight, and then acetonitrile was evaporated under vacuum, and the residual solution was acidified by 1% *v/v* acetic acid and injected for RP-HPLC analysis.

### 3.4. Heterologous Expression in Escherichia coli System

An original recombinant EcAMP1 was obtained as described previously [15]. To make nucleotide-directed mutagenesis, the following oligonucleotides were designed: A1w20aF, cgtcatgaagatgaaccggcgcgtgtgcaggaatgcg; A1w20aR, CGCATTCCTGCACACGCGCCGGTTCATCTTCATGACG; A1DcTermF, taataaaagcttgcggccgcactcga; A1DcTermR, ACGACGGCACTGGCTCACGCATTCC. Reamplification was provided from a based plasmid vector pET32B+EcAMP1.

### 3.5. Cleavage of EcAMP1-X3/X4-Thioredoxin Fusion Protein by Enteropeptidase

A total of 1 mg of freeze-dried fusion proteins were dissolved in 1 mL buffer (50 mM NH_4_HCO_3_ supplied with 1.5 mM CaCl_2_, pH 8.0). A total of 0.5 EU of an enteropeptidase light chain from bovine pancreas (Sigma, Burlington, MA, USA) was dissolved in 10 µL of 1× dilution buffer according to the manufacturer’s recommendations. Cleavage was carried at 37 °C 24 h in the dark. After that, the reaction was stopped by the addition of 100 µL of 0.1% TFA.

### 3.6. Analytical Reversed-Phase HPLC

After proteolysis, products were applied on a Jupiter C_5_ 4.6 × 250 mm column (“Phenomenex”, Torrance, CA, USA) pre-equilibrated by 10% buffer B (80% acetonitrile and 0.1% TFA). The separation was carried out at linear gradient of buffer B (10–50% for 60 min) and a flow rate of 1 mL/min. Detection was carried at 214 nm.

### 3.7. MALDI-TOF MS

Molecular masses of the peptides were measured by a matrix-assisted laser desorption/ionization time-of-flight (MALDI-TOF/TOF) mass spectrometry on an AutoSpeed MALDI-TOF instrument (Bruker Daltonics, Bremen, Germany), in a positive ion mode. 2,5-dihydroxybenzoic acid (Sigma-Aldrich, Ronkonkoma, NY, USA) was applied as a matrix for calibration. Mass spectra were analyzed with the mMass version 5.5.0 software (http://www.mmass.org/).

### 3.8. Molecular Modeling

Modeling of the spatial structure was accomplished using PyMol v. 2.5.2 (DeLano Scientific LLC, USA) software.

### 3.9. Antimicrobial Activity In Vitro

Peptides were tested against opportunistic bacteria and yeasts as well as phytopathogenic filamentous fungi. All tests were carried out using microdilution assay. Bacteria were grown on TSA (trypticase soy agar, BioMérieux, Marcy-l’Étoile, France) plates for 24 h. After that, they were suspended in a standard CA-MHB medium (BBL™ Mueller—Hinton II broth cation-adjusted, Becton Dickinson, Franklin Lakes, New Jersey, USA) up to 1.5 × 10^6^ Colony Forming Units (CFU) mL^−1^. Peptides were diluted to a final concentration of 80–0.625 µM. Cathelicidin (LL37) was applied as a positive control at the same concentration range. Bacterial cultures were incubated with peptides at 37 °C for 48 h. The minimal inhibitory concentration (MIC) of each peptide was determined as the lowest concentration required to inhibit bacterial growth, i.e., to reduce the culture’s OD600 to less than 0.1. OD600 values were measured with a microplate reader (EnVisionTM Multilabel Plate Reader, Perkin Elmer, Waltham, MA, USA). Every tested peptide concentration was assayed in triplicate, and the experiment as a whole was repeated three times [34]. An antiyeast assay was performed in the same way. A YPDA (yeast–peptone–dextrose agar) was used for maintaining the culture and a YPDB (yeast–peptone–dextrose broth) for maintaining the antiyeast assay.

An antifungal assay was performed using PDA (potato–dextrose agar) for maintaining the culture and a PDB (potato–dextrose broth) for a microtiter assay as described previously [35]. Briefly, fungal colonies were grown on solid media at 25 °C for 10 days. Conidia were washed from the surface of mycelium with approximately 10 mL of potato–dextrose broth (Sigma, Burlington, MA, USA) and diluted to a concentration of 10^4^–10^5^ conidia per mL using counting chamber. Fungal suspensions were placed into the wells of microtiter plates (Sigma, Burlington, MA, USA) containing two-fold serial dilutions of each peptide (from 1.0 to 16.0 µM). Incubation with peptides proceeded at 25 °C for 48 h. Inhibition of spore germination was examined by light microscopy using an Axio Scope A1 instrument (Carl Zeiss, Oberkochen, Germany). The degree of inhibition was calculated as the percentage of germinated conidia against their total number. The IC_50_ values were calculated as the peptide concentration that caused a 50% inhibition of spore germination. All experiments were carried out in three replicates.

## 4. Conclusions

This work presents the results of the analysis of structural–functional relationships in α-hairpinin EcAMP1 molecule. This peptide is known to inhibit the growth of filamentous fungi; here it was shown that EcAMP1 possess antiyeast and antibacterial activity against Gram-positive bacteria. Secondary structural integrity, hydrophobicity, and positive surface charge were considered to participate in the antimicrobial activity of the α-hairpinin. Here, it was shown that the modification of all properties assumed led to the antimicrobial activity loss or decrease in the case of fungi.

## Figures and Tables

**Table 1:** Antifungal activity of the native and the truncated forms of EcAMP1 according to a panel of plant pathogenic fungi (IC_50_, µM).

Fungus	EcAMP1-WT	EcAMP1-X1	EcAMP1-X2
*Fusarium oxysporum*	12.9 ± 1.2	15.4 ± 1.1	23.2 ± 2.6
*F. graminearum*	6.8 ± 1.0	9.0 ± 1.4	18.1 ± 2.1
*F. solani*	5.4 ± 1.5	6.9 ± 0.7	11.0 ± 1.9
*Aspergillus niger*	>32.0	>32.0	>32.0
*Bipolaris sorokiniana*	25.7 ± 3.6	>32.0	>32.0
*Alternaria alternata*	18.4 ± 2.7	21.1 ± 2.4	>32.0

**Table 2:** Comparative antifungal activity of the native and the modified forms of the α-hairpinin EcAMP1 (IC50, µM).

Fungus	EcAMP1-WT	EcAMP1-X3	EcAMP1-X4
*F. oxysporum*	9.4 ± 1.4	15.0 ± 2.1	15.8 ± 1.6
*F. graminearum*	5.0 ± 1.1	9.9 ± 1.9	8.5 ± 1.2
*F. solani*	5.6 ± 0.9	8.6 ± 1.5	7.8 ± 0.7

**Table 3:** Variants of the modified EcAMP1 peptides. Cys residues are marked in bold. Amino acid substitution Trp20Ala in EcAMP1-X3 and lacking C-terminal amino acid residues are marked in red.

Peptide Name	Amino Acid Sequence	Modification
EcAMP1-WT	GSGRGS**C**RSQ**C**MRRHEDEPWRVQE**C**VSQ**C**RRRRGGGD	Wild type
EcAMP1-X1	**C**RSQ**C**MRRHEDEPWRVQE**C**VSQ**C**	Truncated form up to outercysteine pair
EcAMP1-X2	**C**MRRHEDEPWRVQE**C**	Truncated form up to innercysteine pair
EcAMP1-X3	GSGRGS**C**RSQ**C**MRRHEDEP**A**RVQE**C**VSQ**C**RRRRGGGD	Trp20Ala substitution
EcAMP1-X4	GSGRGS**C**RSQ**C**MRRHEDEPWRVQE**C**VSQ**C**RR**------**	Remove of six C-terminal amino acid residues

## Data Availability

Not applicable.

## Supplementary Materials

The following supporting information can be downloaded at: https://www.mdpi.com/article/10.3390/molecules27113554/s1. Figure S1, Analytical RP-HPLC profile for EcAMP1-X1 purification. Figure S2, Analytical RP-HPLC profile for EcAMP1-X2 purification. Figure S3, MALDI-TOF mass spectrum for the EcAMP1-X1 peptide. Figure S4, MALDI-TOF mass spectrum for the EcAMP1-X2 peptide. Figure S5, CD curve for the recombinant EcAMP1-WT peptide. Figure S6, Semi-preparative RP-HPLC profile illustrated separation of the recombinant TRX-EcAMP1-X4 fusion protein by L-HEP. Figure S7, Antifungal assays in vitro with optical microscopy detection: control (A,E,I), EcAMP1-WT (B,F,J), EcAMP-X1 (C,G,K) and EcAMP1-X2 (D,H,L). Microphotographs for conidia germination are presented: *Bipolaris sorokiniana* (A–D), *Alternaria alternata* (E–H) and *Fusarium graminearum* (I–L). Table S1, Antifungal activity of the native and the truncated forms of EcAMP1 against *C. albicans*. Table S2, Antibacterial activity of the native and the truncated forms of EcAMP1 against *S. aureus*. Table S3, Antibacterial activity of the native and the truncated forms of EcAMP1 against *E. coli*. Table S4, Antibacterial activity of the native and the truncated forms of EcAMP1 against *P. aeruginosa*.

## Sample Availability

Samples of the compounds are available from the authors.

## References

[1] Hadacek F., Kaufman P.B. (2002). Secondary Metabolites as Plant Traits: Current Assessment and Future Perspectives. CRC. Crit. Rev. Plant Sci..

[2] Zaynab M., Fatima M., Sharif Y., Zafar M.H., Ali H. (2019). Role of primary metabolites in plant defense against pathogens Microbial Pathogenesis Role of primary metabolites in plant defense against pathogens. Microb. Pthogenes..

[3] Zaynab M., Fatima M., Abbas S., Sharif Y., Umair M., Zafar M.H., Bahadar K. (2018). Role of secondary metabolites in plant defense against pathogens. Microb. Pathog..

[4] Egorov T.A., Odintsova T.I., Pukhalsky V.A., Grishin E.V. (2005). Diversity of wheat anti-microbial peptides. Peptides.

[5] Salas C.E., Badillo-Corona J.A., Ramírez-Sotelo G., Oliver-Salvador C. (2015). Biologically active and antimicrobial peptides from plants. Biomed Res. Int..

[6] Tossi A., Sandri L. (2005). Molecular Diversity in Gene-Encoded, Cationic Antimicrobial Polypeptides. Curr. Pharm. Des..

[7] Tam J.P., Wang S., Wong K.H., Tan W.L. (2015). Antimicrobial peptides from plants. Pharmaceuticals.

[8] Kulaeva O., Kliukova M., Afonin A., Sulima A., Zhukov V., Tikhonovich I. (2020). The role of plant antimicrobial peptides (AMPs) in response to biotic and abiotic environmental factors. Biol. Commun..

[9] Slavokhotova A.A., Rogozhin E.A. (2020). Defense Peptides From the α-Hairpinin Family Are Components of Plant Innate Immunity. Front. Plant Sci..

[10] Oparin P.B., Mineev K.S., Dunaevsky Y.E., Arseniev A.S., Belozersky M.A., Grishin E.V., Egorov T.A., Vassilevski A.A. (2012). Buckwheat trypsin inhibitor with helical hairpin structure belongs to a new family of plant defence peptides. Biochem. J..

[11] Yamada K., Shimada T., Kondo M., Nishimura M., Hara-Nishimura I. (1999). Multiple functional proteins are produced by cleaving Asn-Gln bonds of a single precursor by vacuolar processing enzyme. J. Biol. Chem..

[12] Conners R., Konarev A.V., Forsyth J., Lovegrove A., Marsh J., Joseph-Horne T., Shewry P., Brady R.L. (2007). An unusual helix-turn-helix protease inhibitory motif in a novel trypsin inhibitor from seeds of Veronica (*Veronica hederifolia* L.). J. Biol. Chem..

[13] Li F., Yang X.X., Xia H.C., Zeng R., Hu W.G., Li Z., Zhang Z.C. (2003). Purification and characterization of Luffin P1, a ribosome-inactivating peptide from the seeds of Luffa cylindrica. Peptides.

[14] Utkina L.L., Andreev Y.A., Rogozhin E.A., Korostyleva T.V., Slavokhotova A.A., Oparin P.B., Vassilevski A.A., Grishin E.V., Egorov T.A., Odintsova T.I. (2013). Genes encoding 4-Cys antimicrobial peptides in wheat Triticum kiharae Dorof. et Migush.: Multimodular structural organization, instraspecific variability, distribution and role in defence. FEBS J..

[15] Rogozhin E.A., Ryazantsev D.Y., Grishin E.V., Egorov T.A., Zavriev S.K. (2012). Defense peptides from barnyard grass (*Echinochloa crusgalli* L.) seeds. Peptides.

[16] Cui X., Du J., Li J., Wang Z. (2018). Inhibitory site of α-hairpinin peptide from tartary buckwheat has no effect on its antimicrobial activities. Acta Biochim. Biophys. Sin..

[17] Duvick J.P., Rood T., Rao A.G., Marshak D.R. (1992). Purification and characterization of a novel antimicrobial peptide from maize (*Zea mays* L.) kernels. J. Biol. Chem..

[18] Vasilchenko A.S., Yuryev M., Ryazantsev D.Y., Zavriev S.K., Feofanov A.V., Grishin E.V., Rogozhin E.A. (2016). Studying of cellular interaction of hairpin-like peptide EcAMP1 from barnyard grass (*Echinochloa crusgalli* L.) seeds with plant pathogenic fungus Fusarium solani using microscopy techniques. Scanning.

[19] Rogozhin E., Zalevsky A., Mikov A., Smirnov A., Egorov T. (2018). Characterization of hydroxyproline-containing hairpin-like antimicrobial peptide ecamp1-hyp from barnyard grass (*Echinochloa crusgalli* L.) seeds: Structural identification and comparative analysis of antifungal activity. Int. J. Mol. Sci..

[20] Li H., Hu Y., Pu Q., He T., Zhang Q., Wu W., Xia X., Zhang J. (2020). Novel stapling by lysine tethering provides stable and low hemolytic cationic antimicrobial peptides. J. Med. Chem..

[21] Gao J., Zhang M., Zhang F., Wang Y., Ouyang J., Luo X., Yang H., Zhang D., Chen Y., Yu H. (2020). Design of a Sea Snake Antimicrobial Peptide Derivative with Therapeutic Potential against Drug-Resistant Bacterial Infection. ACS Infect. Dis..

[22] Swedberg J.E., Nigon L.V., Reid J.C., de Veer S.J., Walpole C.M., Stephens C.R., Walsh T.P., Takayama T.K., Hooper J.D., Clements J.A. (2009). Substrate-guided design of a potent and selective kallikrein-related peptidase inhibitor for kallikrein 4. Chem. Biol..

[23] Berkut A.A., Usmanova D.R., Peigneur S., Oparin P.B., Mineev K.S., Odintsova T.I., Tytgat J., Arseniev A.S., Grishin E.V., Vassilevski A.A. (2014). Structural Similarity between Defense Peptide from Wheat and Scorpion Neurotoxin Permits Rational Functional Design. J. Biol. Chem..

[24] Tabakmakher V.M., Gigolaev A.M., Peigneur S., Krylov N.A., Tytgat J., Chugunov A.O., Vassilevski A.A., Efremov R.G. (2021). Potassium channel blocker crafted by α-hairpinin scaffold engineering. Biophys. J..

[25] Feurstein C., Meyer V., Jung S. (2022). Structure–Activity Predictions From Computational Mining of Protein Databases to Assist Modular Design of Antimicrobial Peptides. Front. Microbiol..

[26] Bellavita R., Casciaro B., Di Maro S., Brancaccio D., Carotenuto A., Falanga A., Cappiello F., Buommino E., Galdiero S., Novellino E. (2021). First-in-Class Cyclic Temporin L Analogue: Design, Synthesis, and Antimicrobial Assessment. J. Med. Chem..

[27] Nolde S.B., Vassilevski A.A., Rogozhin E.A., Barinov N.A., Balashova T.A., Samsonova O.V., Baranov Y.V., Feofanov A.V., Egorov T.A., Arseniev A.S. (2011). Disulfide-stabilized helical hairpin structure and activity of a novel antifungal peptide EcAMP1 from seeds of barnyard grass (*Echinochloa crus-galli*). J. Biol. Chem..

[28] Frasca L., Lande R. (2012). Role of defensins and cathelicidin LL37 in auto-immune and auto-inflammatory diseases. Curr. Pharm. Biotechnol..

[29] Slavokhotova A.A., Rogozhin E.A., Musolyamov A.K., Andreev Y.A., Oparin P.B., Berkut A.A., Vassilevski A.A., Egorov T.A., Grishin E.V., Odintsova T.I. (2014). Novel antifungal α-hairpinin peptide from Stellaria media seeds: Structure, biosynthesis, gene structure and evolution. Plant Mol. Biol..

[30] Longobardi S., Gravagnuolo A.M., Rea I., De Stefano L., Marino G., Giardina P. (2014). Hydrophobin-coated plates as matrix-assisted laser desorption/ionization sample support for peptide/protein analysis. Anal. Biochem..

[31] Wösten H.A.B., Scholtmeijer K. (2015). Applications of hydrophobins: Current state and perspectives. Appl. Microbiol. Biotechnol..

[32] Sousa D.A., Porto W.F., Silva M.Z., Da Silva T.R., Franco O.L. (2016). Influence of cysteine and tryptophan substitution on DNA-binding activity on maize α-hairpinin antimicrobial peptide. Molecules.

[33] Gunasekera S., Muhammad T., Strömstedt A.A., Rosengren K.J., Göransson U. (2020). Backbone Cyclization and Dimerization of LL-37-Derived Peptides Enhance Antimicrobial Activity and Proteolytic Stability. Front. Microbiol..

[34] Slazak B., Kapusta M., Strömstedt A.A., Słomka A., Krychowiak M., Shariatgorji M., Andrén P.E., Bohdanowicz J., Kuta E., Göransson U. (2018). How Does the Sweet Violet (*Viola odorata* L.) Fight Pathogens and Pests—Cyclotides as a Comprehensive Plant Host Defense System. Front. Plant Sci..

[35] Rogozhin E.A., Slezina M.P., Slavokhotova A.A., Istomina E.A., Korostyleva T.V., Smirnov A.N., Grishin E.V., Egorov T.A., Odintsova T.I. (2015). A novel antifungal peptide from leaves of the weed *Stellaria media* L.. Biochimie.

