# Supplementary material for: Rational Design of Plant Hairpin-like Peptide EcAMP1: Structural–Functional Correlations to Reveal Antibacterial and Antifungal Activity
Source: Molecules. 2022 May 31;27(11):3554. doi: 10.3390/molecules27113554 (PMC9182383; doi:10.3390/molecules27113554)
Supplement: Supplementary file 1 [file molecules-27-03554-s001.zip › molecules-1722688-supplementary.pdf]

Supplementary Material

Rational Design of Plant Hairpin-Like Peptide EcAMP1: Structural–Functional Correlations to Reveal Antibacterial and Antifungal Activity

Anna S. Barashkova [1], Dmitry Yu. Ryazantsev [1] and Eugene A. Rogozhin [1,2,*]

[1] Shemyakin-Ovchinnikov Institute of Bioorganic Chemistry, Russian Academy of Natural Sciences (RAS),
ul. Miklukho-Maklaya, 16/10, 117997 Moscow, Russia; barashkova.an@gmail.com (A.S.B.); d.yu.ryazantsev@gmail.com (D.Y.R.)

[2] Gause Institute of New Antibiotics, ul. Bolshaya Pirogovskaya, 11, 119021 Moscow, Russia

* Correspondence: rea21@list.ru

Print of window 38: Current Chromatogram(s)

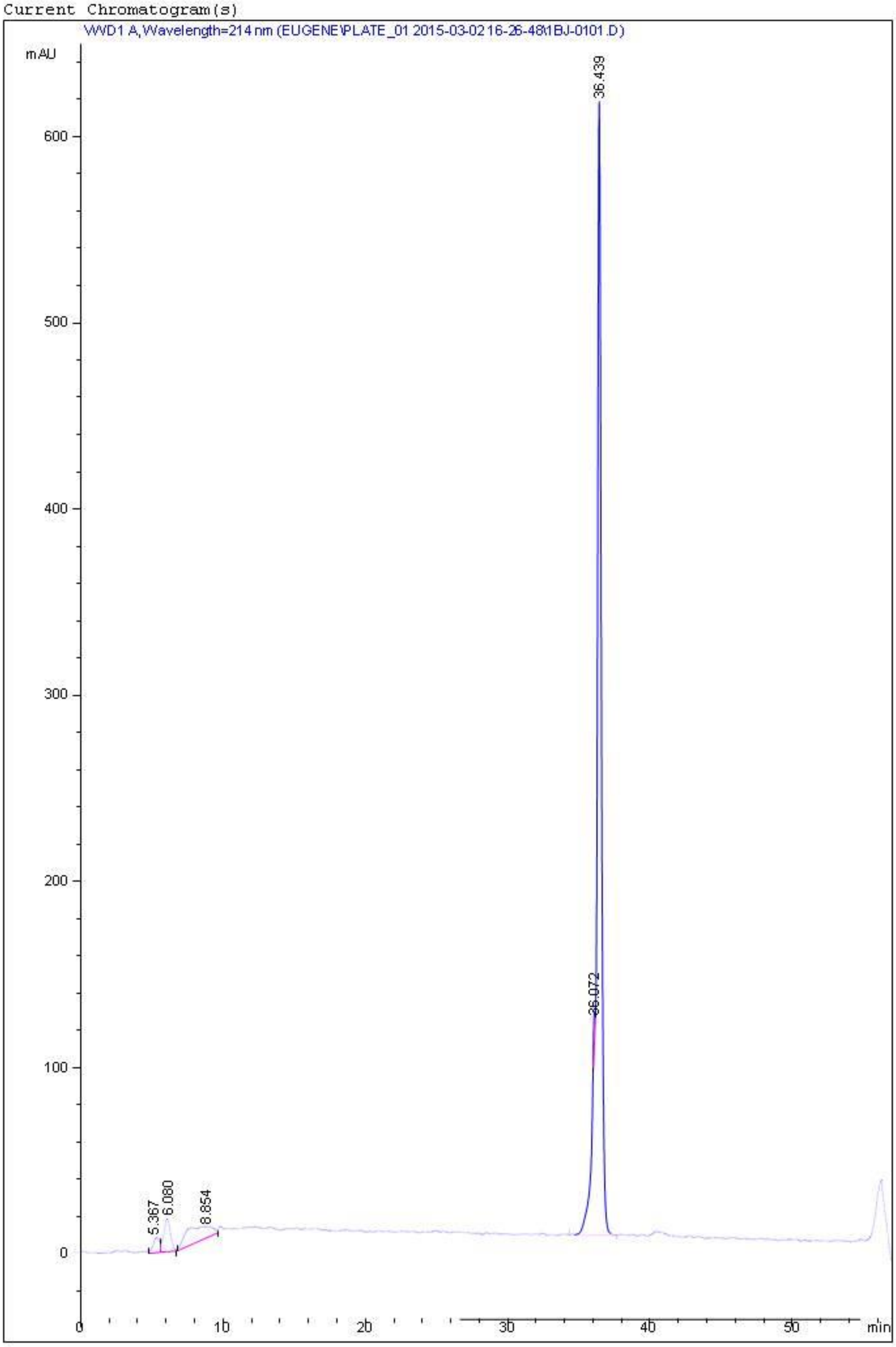

Instrument 1 20.05.2022 15:32:16 Eugen Rogozhin Page 1 of 1

Figure S1 Analytical RP-HPLC profile for EcAMP1-X1 purification. The target peak is eluted at 36.4 min.

Print of window 38: Current Chromatogram(s)

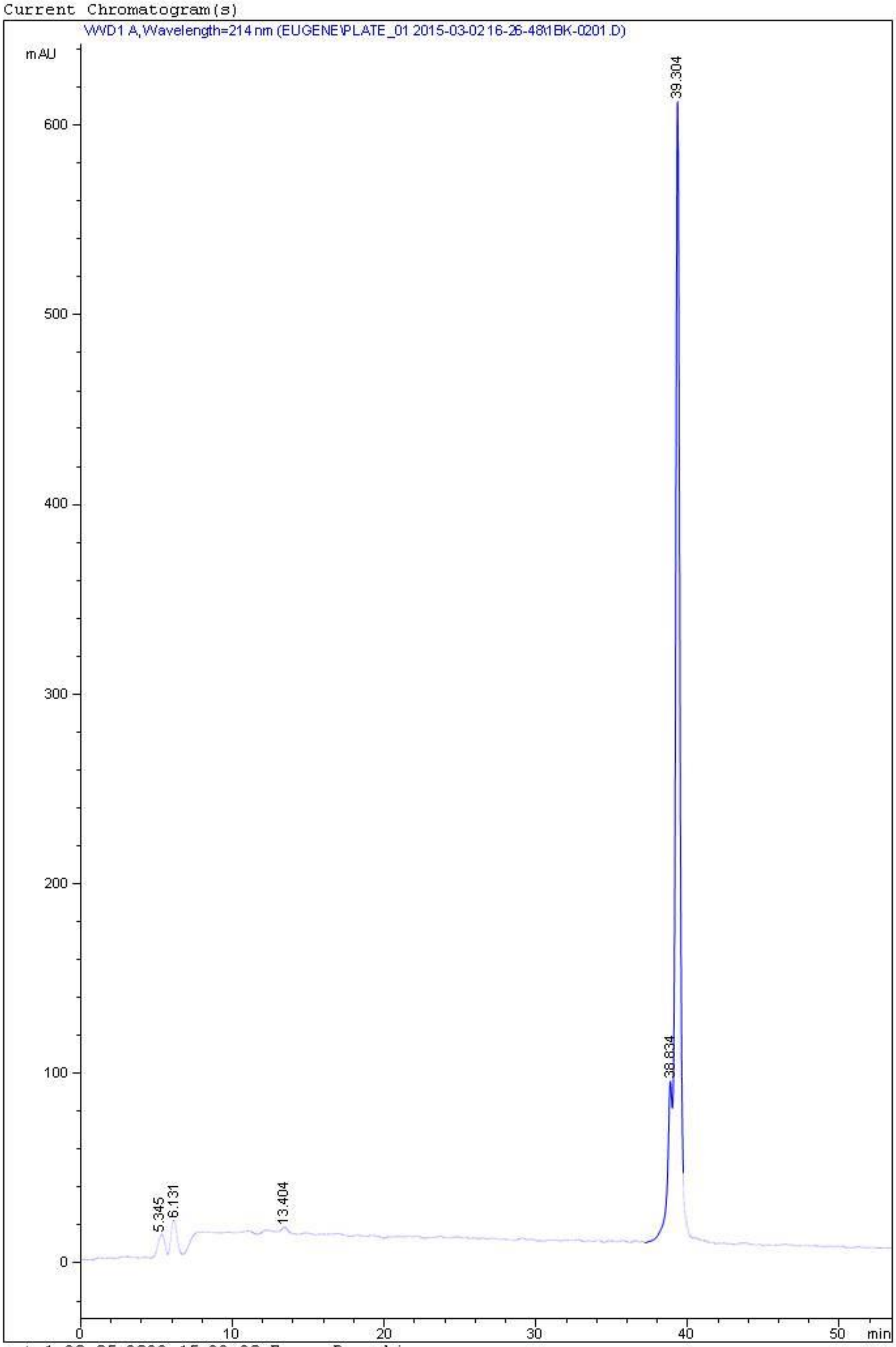

Instrument 1 20.05.2022 15:32:28 Eugen Rogozhin

Figure S2 Analytical RP-HPLC profile for EcAMP1-X2 purification. The target peak is eluted at 39.3 min.

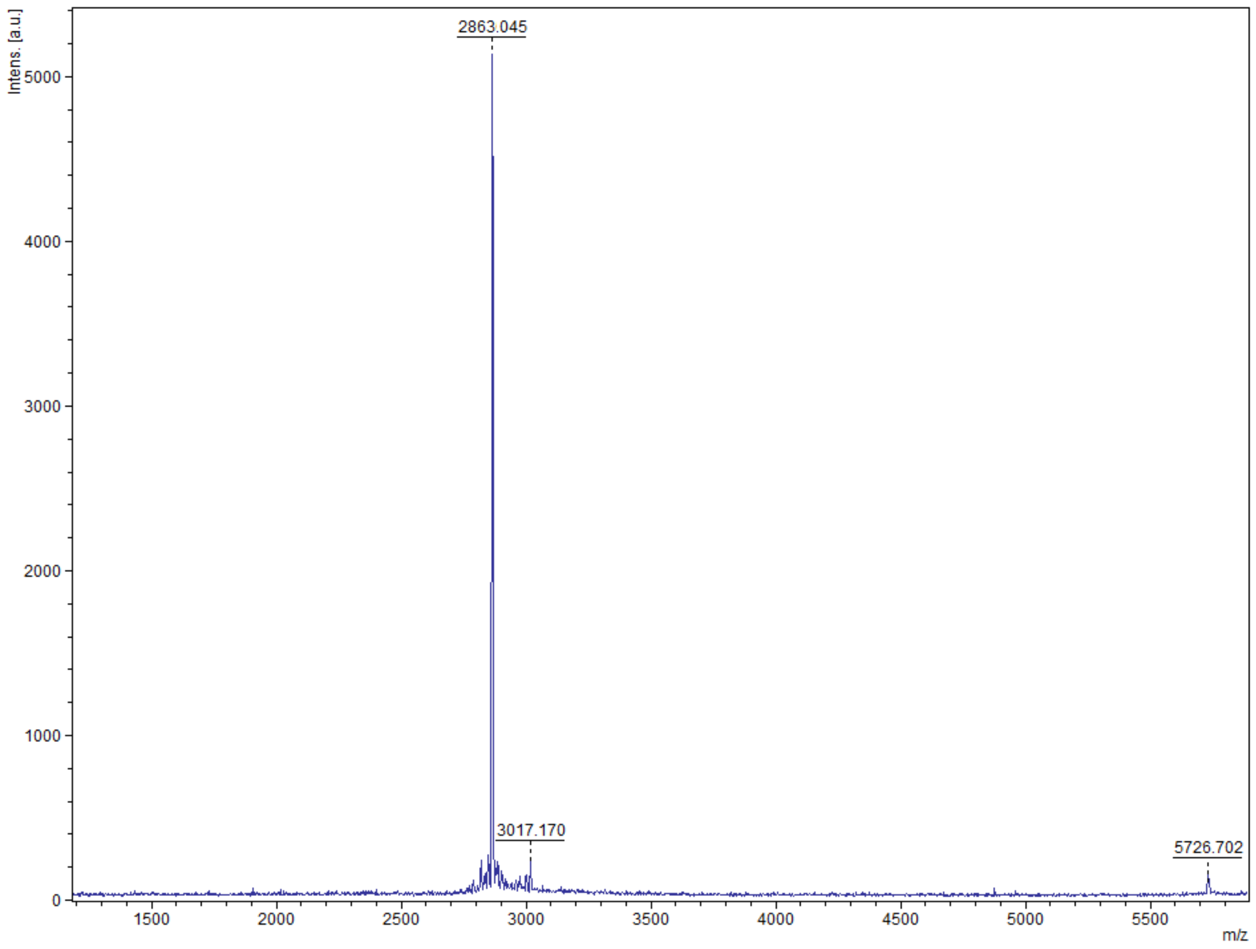

Figure S3 MALDI-TOF mass spectrum for the EcAMP1-X1 peptide. *m*/*z* values are presented in the positive mode.

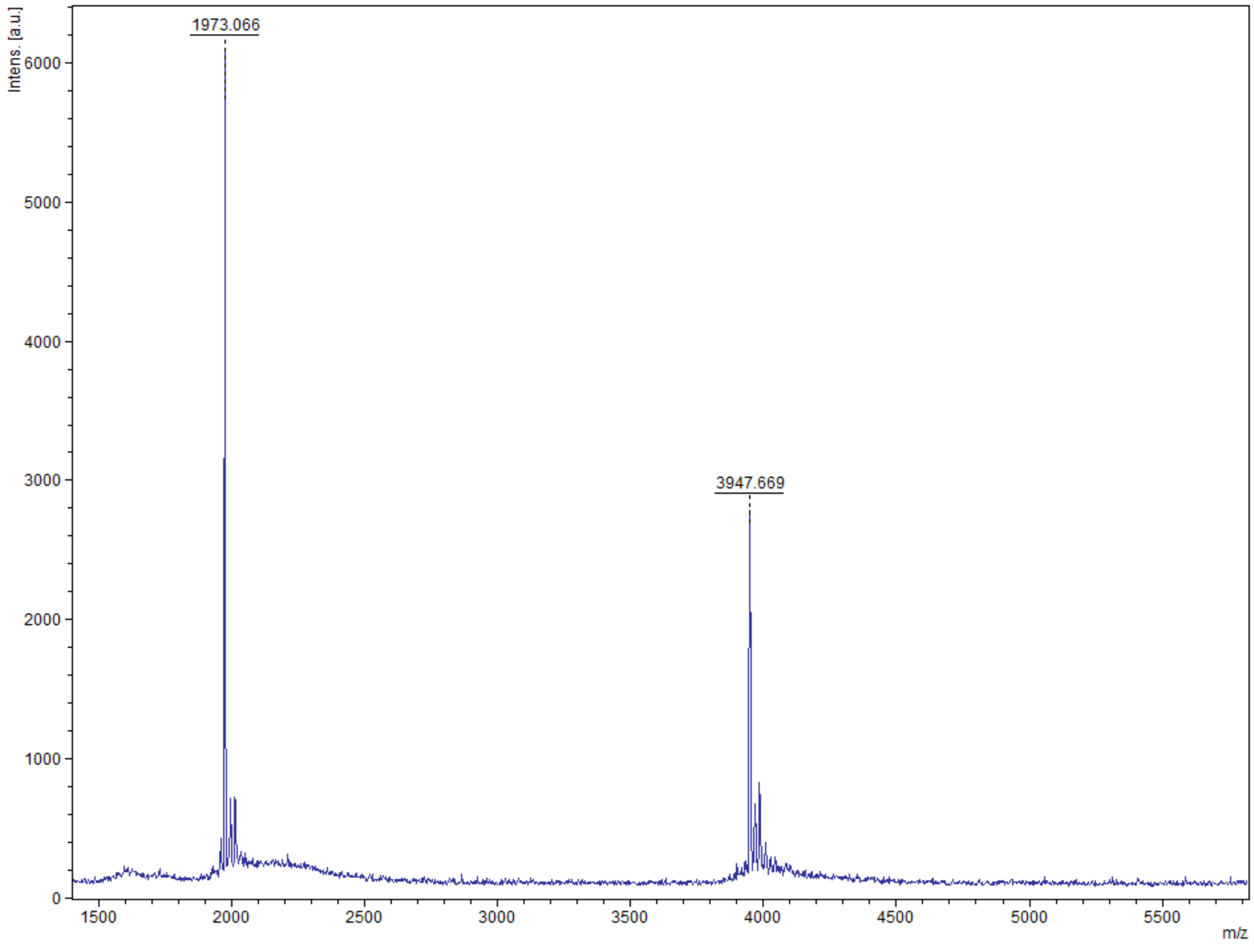

Figure S4 MALDI-TOF mass spectrum for the EcAMP1-X2 peptide. *m*/*z* values are presented in a positive mode.

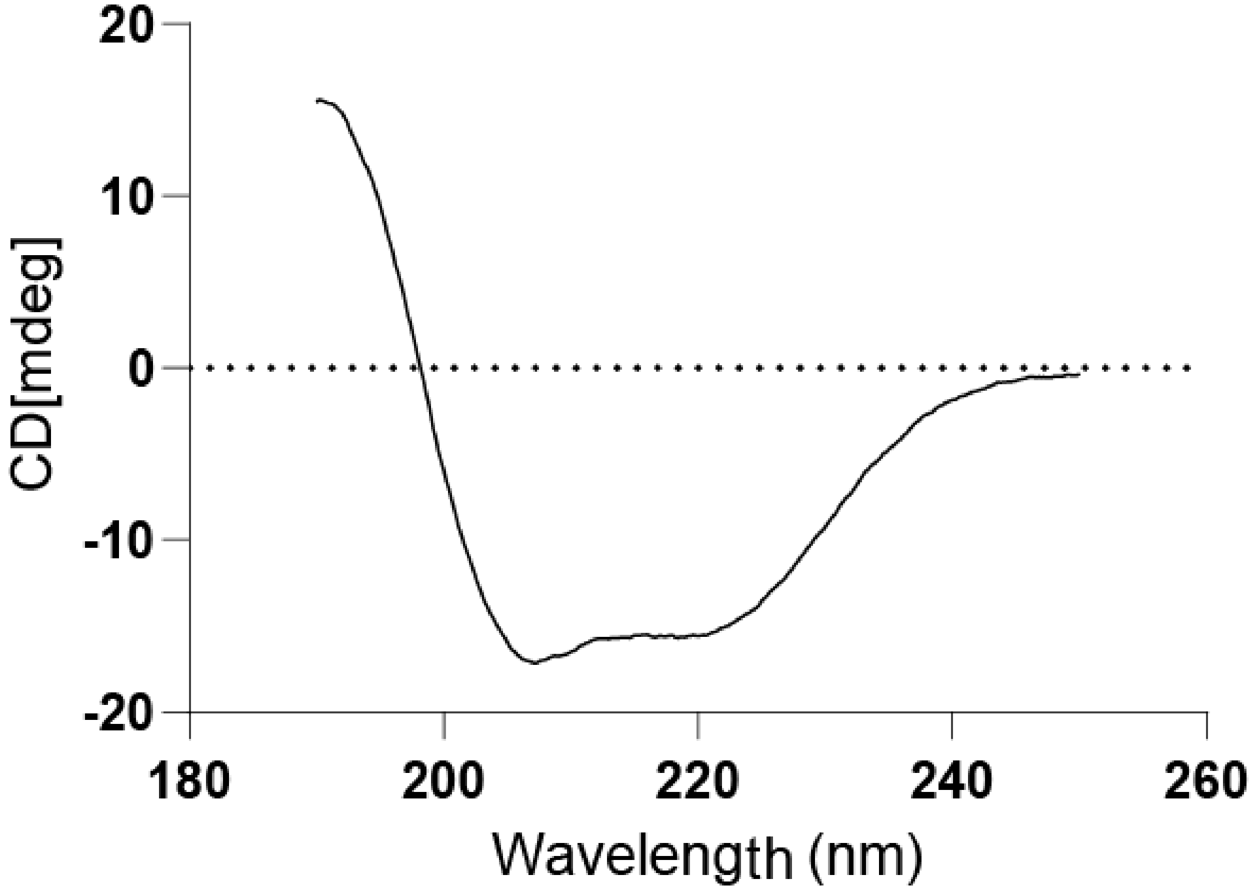

Figure S5 CD curve for the recombinant EcAMP1-WT peptide.

Print of window 38: Current Chromatogram(s)

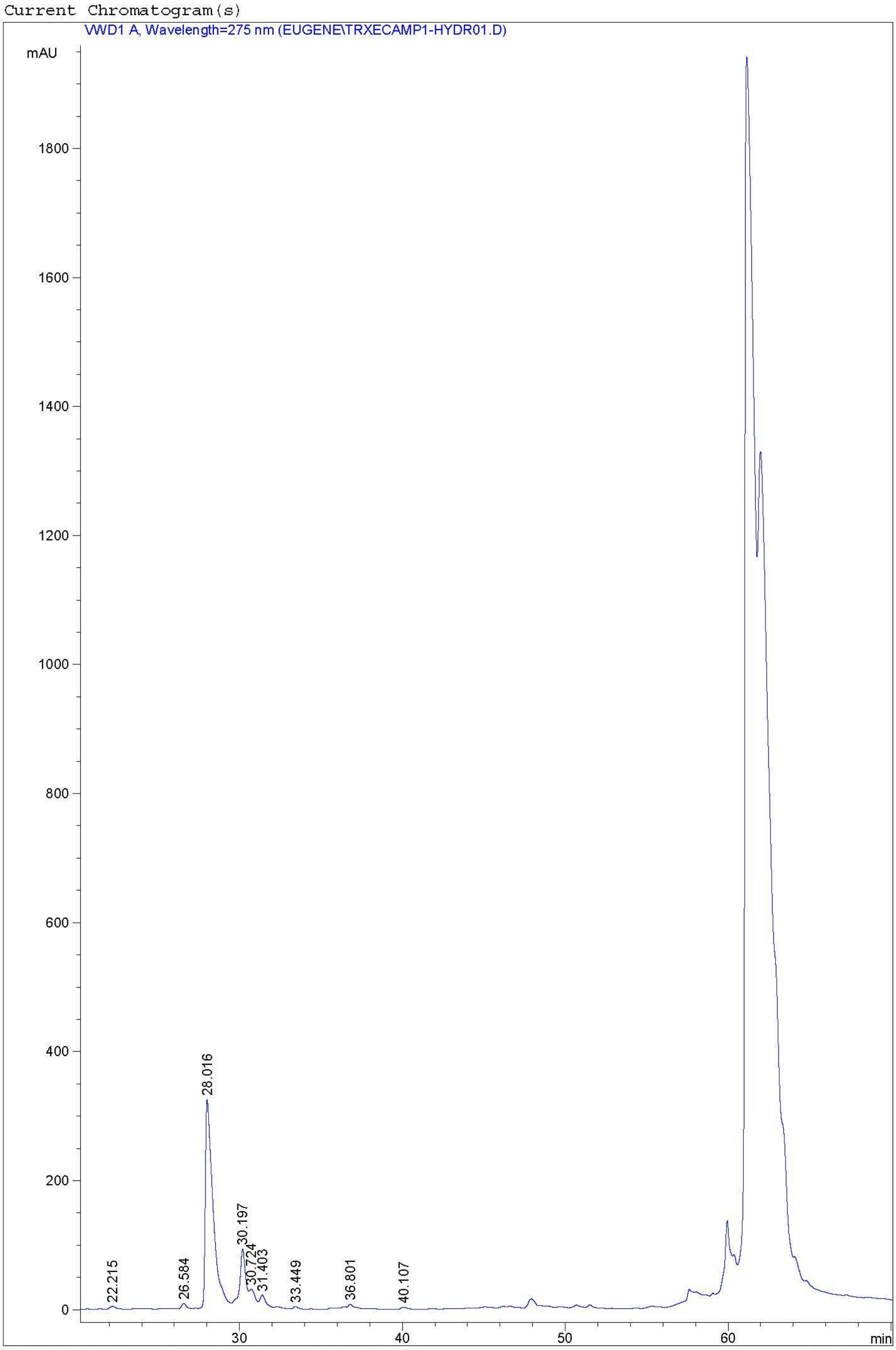

Instrument 1 20.05.2022 15:56:16 Eugen Rogozhin Page 1 of 1

Figure S6 Semi-preparative RP-HPLC profile illustrated separation of the recombinant TRX-EcAMP1-X4 fusion protein by L-HEP. The recombinant EcAMP1-X4 peptide is eluted at 28.0 min.

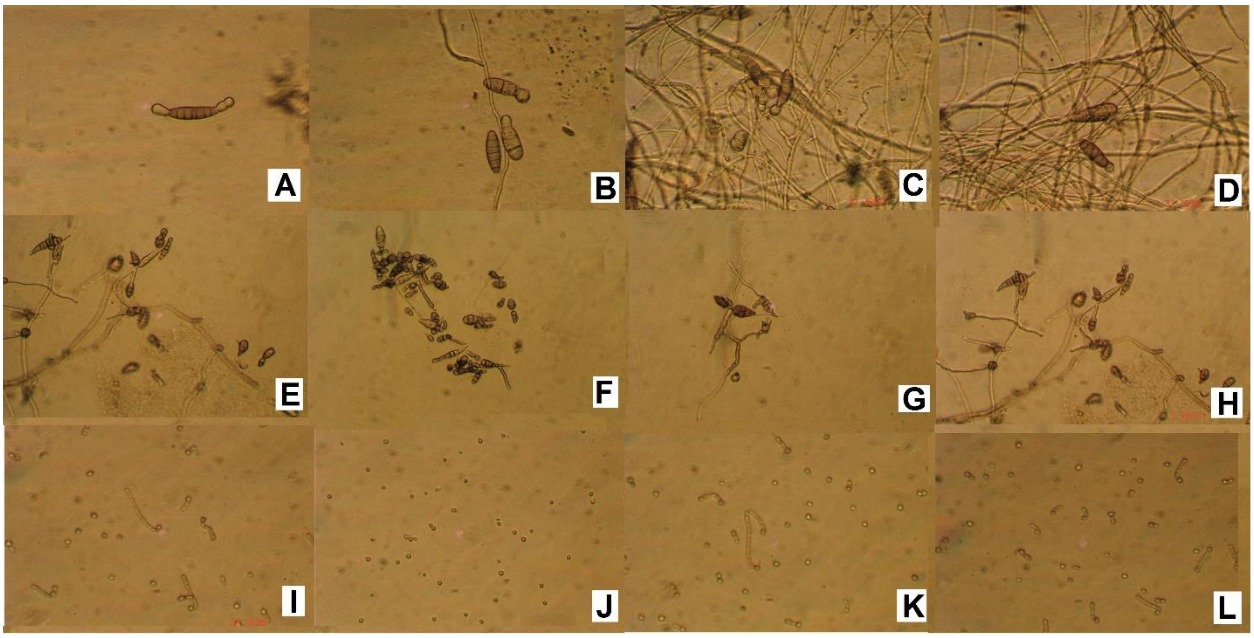

Figure S7 Antifungal assays *in vitro* with optical microscopy detection: control (A, E. I), EcAMP1-WT (B, F, J), EcAMP-X1 (C, G, K) and EcAMP1-X2 (D, H, L). Microphotographs for conidia germination are presented: *Bipolaris sorokiniana* (A-D), *Alternaria alternata* (E-H) and *Fusarium graminearum* (I-L).

Table S1. Antifungal activity of the native and the truncated forms of EcAMP1 against *C. albicans*

Peptide	80µM	40 µM	20 µM	10 µM	5 µM	2.5 µM	1.25 µM	0.625 µM
**EcAMP1-WT**	-	-	-	-	-	-	MIC_{99}	MIC_{50}
**EcAMP1-X1**	+	+	+	+	+	+	+	+
**EcAMP1-X2**	+	+	+	+	+	+	+	+
**LL37**	-	-	-	-	-	-	MIC_{99}	+
**Growth control**	+	+	+	+	+	+	+	+
**Negative control**	-	-	-	-	-	-	-	-

Table S2. Antibacterial activity of the native and the truncated forms of EcAMP1 against *S. aureus*

Peptide	80μM	40 μM	20 μM	10 μM	5 μM	2.5 μM	1.25 μM	0.625 μM
**EcAMP1-WT**	-	-	MIC_{99}	MIC_{95}	MIC_{50}	+	+	+
**EcAMP1-X1**	+	+	+	+	+	+	+	+
**EcAMP1-X2**	+	+	+	+	+	+	+	+
**LL37**	-	-	-	-	-	-	MIC_{99}	MIC_{50}
**Growth control**	+	+	+	+	+	+	+	+
**Negative control**	-	-	-	-	-	-	-	-

Table S3. **Antibacterial activity of the native and the truncated forms of EcAMP1 against *E. coli***

Peptide	80 μM	40 μM	20 μM	10 μM	5 μM	2.5 μM	1.25 μM	0.625 μM
**EcAMP1-WT**	+	+	+	+	+	+	+	+
**EcAMP1-X1**	+	+	+	+	+	+	+	+
**EcAMP1-X2**	+	+	+	+	+	+	+	+
**LL37**	-	-	-	-	-	-	MIC_{99}	+
**Growth control**	+	+	+	+	+	+	+	+
**Negative control**	-	-	-	-	-	-	-	-

Table S4. **Antibacterial activity of the native and the truncated forms of EcAMP1 against *P. aeruginosa***

Peptide	80 μM	40 μM	20 μM	10 μM	5 μM	2.5 μM	1.25 μM	0.625 μM
**EcAMP1-WT**	+	+	+	+	+	+	+	+
**EcAMP1-X1**	+	+	+	+	+	+	+	+
**EcAMP1-X2**	+	+	+	+	+	+	+	+
**LL37**	-	-	-	-	-	-	MIC_{99}	+
**Growth control**	+	+	+	+	+	+	+	+
**Negative control**	-	-	-	-	-	-	-	-
